# Supplementary material for: Evo-SETI: A Mathematical Tool for Cladistics, Evolution, and SETI
Source: Life (Basel). 2017 Apr 6;7(2):18. doi: 10.3390/life7020018 (PMC5492140; doi:10.3390/life7020018)
Supplement: Supplementary file 1 [file life-07-00018-s001.pdf]

#3 APPENDIX

MOLECULAR CLOCK, mathematically derived as ENTROPY of the Running b-Lognormal (RbL) of the Lognormal Process L(t) starting at t=ts and having an arbitrarily assigned mean value m(t).

This is the best result of the Evo-SETI Theory, since it may be extended to Exoplanets and SETI.

Author: Claudio Maccone, e-mail: clmaccon@libero.it
This Maxima file was completed on May 10, 2015.

Clearing the Maxima memory from previous calculations.

(%i1) kill(all);
(%o0) *done*

Preliminary ASSUMPTIONS on the various variables in the game.

(%i1) assume(n>0,t>ts,mu>0,sigma>0,sL>0,te>ts,Ne>0,delta[Ne]>0);
(%o1) $[n>0, t>ts, \mu>0, \sigma>0, sL>0, te>ts, Ne>0, \delta_{Ne}>0]$

(%i2) assume(p>ts);
(%o2) $[p>ts]$

(%i3) depends([m,M],t);
(%o3) $[\mathrm{m}(t), \mathrm{M}(t)]$

(%i4) assume(m(t)>0);
(%o4) $[\mathrm{m}(t)>0]$

(%i5) depends(m,p);
(%o5) $[\mathrm{m}(p)]$

Defining the probability density function (pdf) of the LOGNORMAL stochastic process L(t) starting with probability one at the initial instant t=ts, corresponding to about 3.5 billion years ago on Earth. If the value of 3.8 billion years ago was chosen, all equations would remain just the same, and only slight numeric differences would occur.
In our conventions, past times are denoted by negative values, zero is nowadays, and positive times will be the future on Earth.
Thus, we assume ts = -3.5*10^9*years.

(%i6) def_L_pdf:L_pdf=(%e^(-(log(n)-M(t))^2/(2*(t-ts)*sL^2)))/(sqrt(2*%pi)*n*sqrt(t-ts)*sL);

(%o6) $$L\_pdf = \frac{\%e^{-\frac{(\log(n)-\mathrm{M}(t))^2}{2\,(t-ts)\,sL^2}}}{\sqrt{2}\,\sqrt{\pi}\,n\,\sqrt{t-ts}\,sL}$$

This is equation (18) of the paper.
The independent variable is 0<n<infinity.
The time at which the stochastic process starts is ts.
M(t) is an auxiliary function of the time that will soon be re-expresses in terms of the Mean Value m(t) of the General Lognormal process L(t).
sL (denoted sigma sub L in the paper) is a positive parameter that we will soon determine as a function of the two initial (ts, Ns) and three final (te, Ne delta[Ne]) boundary conditions on L(t).

Checking the normalization condition on the independent variable n.

(%i7) def_normalization_condition:'integrate(rhs(def_L_pdf),n,0,inf)=radcan(integrate(rhs(def_L_pdf),n

(%o7) $$\frac{\int_0^{\infty}\frac{\%e^{-\frac{(\log(n)-\mathrm{M}(t))^2}{2\,(t-ts)\,sL^2}}}{n}\,dn}{\sqrt{2}\,\sqrt{\pi}\,\sqrt{t-ts}\,sL}=1$$

Discovering the MEAN VALUE FORMULA, i.e. the fundamental result of this paper, yielding the mean value m(t) as a function of M(t).
The fact that this integral may be found EXACTLY was a surprise to this author.
It may have a much more profound meaning UNKNOWN to this author at this time.

(%i8) def_mean_value_integral:'integrate(n*rhs(def_L_pdf),n,0,inf)=radcan(integrate(n*rhs(def_L_pdf)

(%o8) $$\frac{\int_0^{\infty}\%e^{-\frac{(\log(n)-\mathrm{M}(t))^2}{2\,(t-ts)\,sL^2}}\,dn}{\sqrt{2}\,\sqrt{\pi}\,\sqrt{t-ts}\,sL}=\%e^{-\frac{(ts-t)\,sL^2-2\,\mathrm{M}(t)}{2}}$$

Defining the MEAN VALUE m(t), yielding the mean value m(t) as a function of M(t).

(%i9) def_mean_value:m(t)=rhs(def_mean_value_integral);

(%o9) $m(t)=\%e^{-\frac{(ts-t)\,sL^2-2\,M(t)}{2}}$

INVERTING the MEAN VALUE FORMULA, i.e. solving it for M(t) as a function of m(t).

(%i10) M_vs_m:distrib(first(solve(log(def_mean_value),M(t))));

(%o10) $M(t)=\frac{(ts-t)\,sL^2}{2}+\log(m(t))$

This formula simplifies at the initial instant ts:

(%i11) M_of_ts_vs_m:subst(ts,t,M_vs_m);

(%o11) $M(ts)=\log(m(ts))$

INITIAL VALUE of both funtions m(t) and M(t) at t=ts.

(%i12) m_of_ts:subst(ts,t,def_mean_value);

(%o12) $m(ts)=\%e^{M(ts)}$

(%i13) def_Ns:Ns=m(ts);

(%o13) $Ns=m(ts)$

(%i14) Ns_vs_M_of_ts:def_Ns,m_of_ts;

(%o14) $Ns=\%e^{M(ts)}$

(%i15) def_Ns_vs_m_of_ts:first(solve(def_Ns,m(ts)));

(%o15) $m(ts)=Ns$

Descriptive Statistics of the General Lognormal Process L(t)

Finding the k-th MOMENT of the General Lognormal pdf of L(t),
i.e. finding ALL MOMENTS of L(t).
With k=0 we get the normalization condition of L(t) again.
With k=1 we get the mean value m(t) of L(t) again.
With k=2 we get the mean value of the square of L(t).
And so on.

(%i16) declare(k,integer);
(%o16) *done*

Defining and finding ALL MOMENTS.

(%i17) def_kth_moment:'integrate(n^k*rhs(def_L_pdf),n,0,inf)=radcan(integrate(n^k*rhs(def_L_pdf),n,0

Is k positive, negative or zero? p;
Is k-1 positive, negative or zero? p;

(%o17) $$\frac{\int_0^{\infty} n^{k-1}\, \%e^{-\frac{(\log(n)-\mathrm{M}(t))^2}{2\,(t-ts)\,sL^2}}\, dn}{\sqrt{2}\,\sqrt{\pi}\,\sqrt{t-ts}\,sL}=\%e^{-\frac{(k^2\,ts-k^2\,t)\,sL^2-2\,k\,\mathrm{M}(t)}{2}}$$

(%i18) kth_moment:rhs(def_kth_moment),M_vs_m;

(%o18) $$\%e^{-\frac{(k^2\,ts-k^2\,t)\,sL^2-2\,k\left(\frac{(ts-t)\,sL^2}{2}+\log(\mathrm{m}(t))\right)}{2}}$$

(%i19) def_OK_kth_moment:'OK_kth_moment=expand(factor(radcan(kth_moment)));

(%o19) $$OK\_kth\_moment=\mathrm{m}(t)^k\,\%e^{-\frac{k^2\,ts\,sL^2}{2}+\frac{k\,ts\,sL^2}{2}+\frac{k^2\,t\,sL^2}{2}-\frac{k\,t\,sL^2}{2}}$$

(%i20) def_Final_kth_moment:Final_kth_moment=m(t)^k*%e^(k*(k-1)*(t-ts)*sL^2/2);

(%o20) $$Final\_kth\_moment=\mathrm{m}(t)^k\,\%e^{\frac{(k-1)\,k\,(t-ts)\,sL^2}{2}}$$

(%i21) radcan(expand(def_OK_kth_moment-def_Final_kth_moment));

(%o21) $$OK\_kth\_moment-Final\_kth\_moment=0$$

Second Moment, i.e. mean value of the square of L(t), or, if you so prefer,
<[L(t)]^2>.

(%i22) def_mean_value_of_the_square:subst(2,k,rhs(def_Final_kth_moment));

(%o22) $$\mathrm{m}(t)^2\,\%e^{(t-ts)\,sL^2}$$

VARIANCE of L(t) as a function of t.

(%i23) variance_of_m:def_mean_value_of_the_square-m(t)^2;

(%o23) $\mathrm{m}(t)^2\,\%\mathrm{e}^{(t-ts)\,sL^2}-\mathrm{m}(t)^2$

(%i24) OK_variance:factor(variance_of_m);

(%o24) $-\mathrm{m}(t)^2\,\%\mathrm{e}^{-ts\,sL^2}\left(\%\mathrm{e}^{ts\,sL^2}-\%\mathrm{e}^{t\,sL^2}\right)$

(%i25) def_variance_with_the_coefficient_of_variation:m(t)^2*(%e^((t-ts)*sL^2)-1);

(%o25) $\mathrm{m}(t)^2\left(\%\mathrm{e}^{(t-ts)\,sL^2}-1\right)$

(%i26) radcan(OK_variance-def_variance_with_the_coefficient_of_variation);

(%o26) 0

STANDARD DEVIATION OF L(t) as a function of t: St_Dev(t)

(%i27) def_St_Dev:St_Dev=sqrt(def_variance_with_the_coefficient_of_variation);

(%o27) $St\_Dev=\mathrm{m}(t)\sqrt{\%\mathrm{e}^{(t-ts)\,sL^2}-1}$

FINDING sL as expressed in terms of the initial (ts, Ns) and final (te,Ne,delta[Ne]) inputs.

(%i28) at_end:delta[Ne]=subst(te,t,rhs(def_St_Dev));

(%o28) $\delta_{Ne}=\mathrm{m}(te)\sqrt{\%\mathrm{e}^{(te-ts)\,sL^2}-1}$

(%i29) def_Ne_vs_m_of_ts:m(te)=Ne;

(%o29) $\mathrm{m}(te)=Ne$

(%i30) sL_square_from_boundary_inputs:first(solve(at_end^2,sL^2)),def_Ne_vs_m_of_ts;

(%o30) $sL^2=-\dfrac{\log\left(\dfrac{\delta_{Ne}^2}{Ne^2}+1\right)}{ts-te}$

(%i31) sL_from_boundary_inputs:sqrt(sL_square_from_boundary_inputs);

(%o31) $sL=\dfrac{\sqrt{\log\left(\dfrac{\delta_{Ne}^2}{Ne^2}+1\right)}}{\sqrt{te-ts}}$

Upper Standard Deviation_CURVE.

(%i32) u_st_dev_curve(t):=m(t)*(1+sqrt(%e^(sL^2*(t-ts))-1));

(%o32) $\mathrm{u\_st\_dev\_curve}(t):=\mathrm{m}(t)\left(1+\sqrt{\%e^{sL^2\,(t-ts)}-1}\right)$

(%i33) u_st_dev_curve(ts);

(%o33) $\mathrm{m}(ts)$

Expressing sL^2 in terms of the Boundary Conditions.

(%i34) first_form_of_Upper_St_Dev_Curve:subst(rhs(sL_square_from_boundary_inputs),sL^2,u_st_dev

(%o34) $\mathrm{m}(t)\left(\sqrt{\%e^{-\frac{\log\left(\frac{\delta_{Ne}^2}{Ne^2}+1\right)(t-ts)}{ts-te}}-1}+1\right)$

Easier form of the above result.

(%i35) easy_form_of_Upper_St_Dev_Curve:m(t)*(sqrt((delta[Ne]^2/Ne^2+1)^((t-ts)/(te-ts))-1)+1);

(%o35) $\mathrm{m}(t)\left(\sqrt{\left(\frac{\delta_{Ne}^2}{Ne^2}+1\right)^{\frac{t-ts}{te-ts}}-1}+1\right)$

(%i36) radcan(first_form_of_Upper_St_Dev_Curve-easy_form_of_Upper_St_Dev_Curve);

(%o36) 0

Final form of the Upper Standard Deviation Curve.

(%i37) Upper_St_Dev_Curve(t):=m(t)*(sqrt((delta[Ne]^2/Ne^2+1)^((t-ts)/(te-ts))-1)+1);

(%o37) $\mathrm{Upper\_St\_Dev\_Curve}(t):=\mathrm{m}(t)\left(\sqrt{\left(\frac{\delta_{Ne}^2}{Ne^2}+1\right)^{\frac{t-ts}{te-ts}}-1}+1\right)$

(%i38) def_USDC:USDC=m(t)*(sqrt((delta[Ne]^2/Ne^2+1)^((t-ts)/(te-ts))-1)+1);

(%o38) $USDC=\mathrm{m}(t)\left(\sqrt{\left(\frac{\delta_{Ne}^2}{Ne^2}+1\right)^{\frac{t-ts}{te-ts}}-1}+1\right)$

Checking that Upper Standard Deviation Curve at te = Ne + delta[Ne]

(%i39) subst(te,t,easy_form_of_Upper_St_Dev_Curve);

(%o39) $\left(\frac{\delta_{Ne}}{Ne}+1\right)\mathrm{m}(te)$

(%i40) %,def_Ne_vs_m_of_ts;

(%o40) $Ne\left(\frac{\delta_{Ne}}{Ne}+1\right)$

(%i41) expand(%);

(%o41) $\delta_{Ne}+Ne$

Lower Standard Deviation CURVE.

(%i42) l_st_dev_curve(t):=m(t)*(1-sqrt(%e^(sL^2*(t-ts))-1));

(%o42) $\mathrm{l\_st\_dev\_curve}(t):=\mathrm{m}(t)\left(1-\sqrt{\%e^{sL^2\,(t-ts)}-1}\right)$

(%i43) u_st_dev_curve(ts);

(%o43) $\mathrm{m}(ts)$

Expressing sL^2 in terms of the Boundary Conditions.

(%i44) first_form_of_Lower_St_Dev_Curve:subst(rhs(sL_square_from_boundary_inputs),sL^2,l_st_dev_

(%o44) $\mathrm{m}(t)\left(1-\sqrt{\%e^{-\frac{\log\left(\frac{\delta_{Ne}^2}{Ne^2}+1\right)(t-ts)}{ts-te}}-1}\right)$

Easier form of the above result.

(%i45) easy_form_of_Lower_St_Dev_Curve:m(t)*(1-sqrt((delta[Ne]^2/Ne^2+1)^((t-ts)/(te-ts))-1));

(%o45) $\mathrm{m}(t)\left(1-\sqrt{\left(\frac{\delta_{Ne}^2}{Ne^2}+1\right)^{\frac{t-ts}{te-ts}}-1}\right)$

(%i46) radcan(first_form_of_Lower_St_Dev_Curve-easy_form_of_Lower_St_Dev_Curve);

(%o46) 0

Final form of the Lower Standard Deviation Curve.

(%i47) Lower_St_Dev_Curve(t):=m(t)*(1-sqrt(%e^(-((t-ts)*log(delta[Ne]^2/Ne^2+1))/(ts-te))-1));

(%o47) $\mathrm{Lower\_St\_Dev\_Curve}(t):=\mathrm{m}(t)\left(1-\sqrt{\%e^{\frac{-(t-ts)\log\left(\frac{\delta_{Ne}^2}{Ne^2}+1\right)}{ts-te}}-1}\right)$

(%i48) def_LSDC:LSDC=m(t)*(1-sqrt(%e^(-((t-ts)*log(delta[Ne]^2/Ne^2+1))/(ts-te))-1));

(%o48) $LSDC=\mathrm{m}(t)\left(1-\sqrt{\%e^{-\frac{\log\left(\frac{\delta_{Ne}^2}{Ne^2}+1\right)(t-ts)}{ts-te}}-1}\right)$

Easier form of the above result.

(%i49) easy_form_of_Lower_St_Dev_Curve:m(t)*(1-sqrt((delta[Ne]^2/Ne^2+1)^((t-ts)/(te-ts))-1));

(%o49) $\mathrm{m}(t)\left(1-\sqrt{\left(\frac{\delta_{Ne}^2}{Ne^2}+1\right)^{\frac{t-ts}{te-ts}}-1}\right)$

(%i50) radcan(first_form_of_Lower_St_Dev_Curve-easy_form_of_Lower_St_Dev_Curve);

(%o50) 0

Checking that Lower Standard Deviation Curve at te = Ne - delta[Ne]

(%i51) subst(te,t,easy_form_of_Lower_St_Dev_Curve);

(%o51) $\left(1-\frac{\delta_{Ne}}{Ne}\right)\mathrm{m}(te)$

(%i52) %,def_Ne_vs_m_of_ts;

(%o52) $Ne\left(1-\frac{\delta_{Ne}}{Ne}\right)$

(%i53) expand(%);

(%o53) $Ne-\delta_{Ne}$

MEDIAN (= fifty-fifty probability) as a function of m(t).

(%i54) assume(median>0);

(%o54) $[median>0]$

(%i55) def_median:'integrate(rhs(def_L_pdf),n,0,median)=radcan(integrate(rhs(def_L_pdf),n,0,median));

$$(\%o55)\ \frac{\int_0^{median} \frac{\%e^{-\frac{(\log(n)-M(t))^2}{2\,(t-ts)\,sL^2}}}{n}\,dn}{\sqrt{2}\,\sqrt{\pi}\,\sqrt{t-ts}\,sL}=\frac{\mathrm{erf}\left(\frac{(\sqrt{2}\,M(t)-\sqrt{2}\,\log(median))\,\sqrt{t-ts}}{(2\,ts-2\,t)\,sL}\right)+1}{2}$$

(%i56) median_eq:first(solve(rhs(def_median)=1/2,median));

$$(\%o56)\ median=\%e^{M(t)}$$

(%i57) median_vs_m:median_eq,M_vs_m;

$$(\%o57)\ median=m(t)\,\%e^{\frac{(ts-t)\,sL^2}{2}}$$

MODE, i.e. PEAK ABSCISSA as a function of m(t).

(%i58) peak_abscissa:first(solve(diff(rhs(def_L_pdf),n)=0,n));

$$(\%o58)\ n=\%e^{ts\,sL^2-t\,sL^2+M(t)}$$

(%i59) M_vs_m;

$$(\%o59)\ M(t)=\frac{(ts-t)\,sL^2}{2}+\log(m(t))$$

(%i60) peak_abscissa_with_m:peak_abscissa,M_vs_m;

$$(\%o60)\ n=m(t)\,\%e^{\frac{(ts-t)\,sL^2}{2}+ts\,sL^2-t\,sL^2}$$

(%i61) final_peak_abscissa:radcan(peak_abscissa_with_m);

$$(\%o61)\ n=m(t)\,\%e^{\frac{(3\,ts-3\,t)\,sL^2}{2}}$$

Ordinate of the peak = Mode Ordinate

(%i62) def_L_pdf;

$$(\%o62)\ L\_pdf=\frac{\%e^{-\frac{(\log(n)-M(t))^2}{2\,(t-ts)\,sL^2}}}{\sqrt{2}\,\sqrt{\pi}\,n\,\sqrt{t-ts}\,sL}$$

(%i63) L_pdf_with_m:def_L_pdf,M_vs_m;

(%o63) $L\_pdf = \frac{\%e^{-\frac{\left(-\frac{(ts-t)\, sL^2}{2} - \log(m(t)) + \log(n)\right)^2}{2\,(t-ts)\, sL^2}}}{\sqrt{2}\,\sqrt{\pi}\, n\,\sqrt{t-ts}\, sL}$

(%i64) L_pdf_with_m,final_peak_abscissa;

(%o64) $L\_pdf = \frac{\%e^{-\frac{\left(\log\left(m(t)\,\%e^{\frac{(3\,ts-3\,t)\, sL^2}{2}}\right) - \frac{(ts-t)\, sL^2}{2} - \log(m(t))\right)^2}{2\,(t-ts)\, sL^2} - \frac{(3\,ts-3\,t)\, sL^2}{2}}}{\sqrt{2}\,\sqrt{\pi}\, m(t)\,\sqrt{t-ts}\, sL}$

(%i65) radcan(%);

(%o65) $L\_pdf = \frac{\%e^{(t-ts)\, sL^2}}{\sqrt{2}\,\sqrt{\pi}\, m(t)\,\sqrt{t-ts}\, sL}$

Proving the PEAK-LOCUS THEOREM for an arbitrarily assigned Mean Value m(t)

Recalling the peak abscissa and ordinate of any b-lognormal.

(%i66) from_L_bln_to_RbL:[n=t-b,M(t)=mu,sL=sqrt(sigma^2/(t-ts))];

(%o66) $[n = t-b, M(t) = \mu, sL = \frac{\sigma}{\sqrt{t-ts}}]$

(%i67) def_RbL:RbL_pdf=rhs(def_L_pdf),from_L_bln_to_RbL;

(%o67) $RbL\_pdf = \frac{\%e^{-\frac{(\log(t-b)-\mu)^2}{2\,\sigma^2}}}{\sqrt{2}\,\sqrt{\pi}\,\sigma\,(t-b)}$

(%i68) RbL_maximum:first(solve(diff(rhs(def_RbL),t)=0,t));

(%o68) $t = \%e^{\mu-\sigma^2} + b$

(%i69) def_RbL_peak_abscissa:subst(p,t,RbL_maximum);

(%o69) $p = \%e^{\mu-\sigma^2} + b$

(%i70) finding_the_RbL_peak_ordinate:def_RbL,RbL_maximum;

(%o70) $RbL\_pdf = \frac{\%e^{\frac{\sigma^2}{2}-\mu}}{\sqrt{2}\sqrt{\pi}\,\sigma}$

(%i71) def_RbL_peak_ordinate:P=rhs(finding_the_RbL_peak_ordinate);

(%o71) $P = \frac{\%e^{\frac{\sigma^2}{2}-\mu}}{\sqrt{2}\sqrt{\pi}\,\sigma}$

(%i72) def_RbL_peak_coordinates:[def_RbL_peak_abscissa,def_RbL_peak_ordinate];

(%o72) $[p = \%e^{\mu-\sigma^2}+b, P = \frac{\%e^{\frac{\sigma^2}{2}-\mu}}{\sqrt{2}\sqrt{\pi}\,\sigma}]$

Equalling the peak value of the Running b-Lognormal to the normalized_m_of_p (i.e. the L mean value).

(%i73) def_RbL_peak_equals_L_mean_value:rhs(second(def_RbL_peak_coordinates))=subst(p,t,rhs(def_

(%o73) $\frac{\%e^{\frac{\sigma^2}{2}-\mu}}{\sqrt{2}\sqrt{\pi}\,\sigma} = \%e^{-\frac{(ts-p)\, sL^2 - 2\,\mathrm{M}(p)}{2}}$

SEPARATING the two equations. This is the KEY mathematical step to find mu and sigma of the RbL.

(%i74) Two_eqs:[%e^(sigma^2/2-mu)=%e^((p*sL^2)/2),1/(sqrt(2)*sqrt(%pi)*sigma)=%e^(-ts*sL^2/2+M

(%o74) $[\%e^{\frac{\sigma^2}{2}-\mu} = \%e^{\frac{p\ sL^2}{2}}, \frac{1}{\sqrt{2}\sqrt{\pi}\,\sigma} = \%e^{\mathrm{M}(p)-\frac{ts\ sL^2}{2}}]$

Checking that the two separated equation, when multiplied, yield the original single equation.

(%i75) Check:radcan(first(Two_eqs)*second(Two_eqs)-def_RbL_peak_equals_L_mean_value);

(%o75) $0=0$

SOLVING the second equation for sigma(p). This yields the sigma of M(p) equation.

(%i76) sigma_of_M_of_p:first(solve(second(Two_eqs),sigma));

(%o76) $\sigma = \frac{\%e^{\frac{ts\ sL^2}{2}-\mathrm{M}(p)}}{\sqrt{2}\sqrt{\pi}}$

In the last equation, let us replace M(p) by its expression in terms of m(p).

(%i77) sigma_of_m_of_p:sigma_of_M_of_p,subst(p,t,M_vs_m);

(%o77) $$\sigma=\frac{\%e^{\frac{ts\, sL^2}{2}-\frac{(ts-p)\, sL^2}{2}}}{\sqrt{2}\,\sqrt{\pi}\,\mathrm{m}(p)}$$

So, the final sigma of m(p) is found.

(%i78) sigma_of_p:radcan(sigma_of_m_of_p);

(%o78) $$\sigma=\frac{\%e^{\frac{p\, sL^2}{2}}}{\sqrt{2}\,\sqrt{\pi}\,\mathrm{m}(p)}$$

SOLVING the first equation for mu(p).

(%i79) towards_mu_of_p:distrib(first(solve(first(Two_eqs),mu)));

(%o79) $$\mu=\frac{\sigma^2}{2}-\frac{p\, sL^2}{2}$$

SUBSTITUTING sigma(p) into the last equation for mu(p).

(%i80) mu_of_p:towards_mu_of_p,sigma_of_p;

(%o80) $$\mu=\frac{\%e^{p\, sL^2}}{4\,\pi\,\mathrm{m}(p)^2}-\frac{p\, sL^2}{2}$$

(%i81) final_sigma_and_mu_of_m_of_p:[sigma_of_p,mu_of_p];

(%o81) $$[\sigma=\frac{\%e^{\frac{p\, sL^2}{2}}}{\sqrt{2}\,\sqrt{\pi}\,\mathrm{m}(p)},\mu=\frac{\%e^{p\, sL^2}}{4\,\pi\,\mathrm{m}(p)^2}-\frac{p\, sL^2}{2}]$$

Checking the truth of the Peak-Locus Theorem by replacing these
mu and sigma into the Peak Ordinate of the Running b-Lognormal.

(%i82) def_RbL_peak_ordinate,final_sigma_and_mu_of_m_of_p;

(%o82) $P=\mathrm{m}(p)$

Particular cases of the Peak-Locus Theorem

tsGBM case.

(%i83) assume(A>0,B>0);

(%o83) $[A>0, B>0]$

(%i84) tsGBM_case:[m(p)=m(ts)*%e^(B*(p-ts)),sL=sqrt(2*B)];

(%o84) $[\mathrm{m}(p)=\mathrm{m}(ts)\,\%e^{(p-ts)\,B}, sL=\sqrt{2}\,\sqrt{B}]$

(%i85) towards_final_tsGBM_sigma_and_mu_of_p:final_sigma_and_mu_of_m_of_p,tsGBM_case;

(%o85) $[\sigma=\frac{\%e^{p\,B-(p-ts)\,B}}{\sqrt{2}\,\sqrt{\pi}\,\mathrm{m}(ts)}, \mu=\frac{\%e^{2\,p\,B-2\,(p-ts)\,B}}{4\,\pi\,\mathrm{m}(ts)^2}-p\,B]$

(%i86) final_tsGBM_sigma_and_mu_of_p:distrib(radcan(towards_final_tsGBM_sigma_and_mu_of_p));

(%o86) $[\sigma=\frac{\%e^{ts\,B}}{\sqrt{2}\,\sqrt{\pi}\,\mathrm{m}(ts)}, \mu=\frac{\%e^{2\,ts\,B}-4\,\pi\,p\,\mathrm{m}(ts)^2\,B}{4\,\pi\,\mathrm{m}(ts)^2}]$

Finding the numeric value of B.

(%i87) tsGBM_case_at_te:subst(te,p,tsGBM_case);

(%o87) $[\mathrm{m}(te)=\mathrm{m}(ts)\,\%e^{(te-ts)\,B}, sL=\sqrt{2}\,\sqrt{B}]$

(%i88) B_of_tsGBM:first(solve(first(tsGBM_case_at_te),B));

(%o88) $B=-\frac{\log\left(\frac{\mathrm{m}(te)}{\mathrm{m}(ts)}\right)}{ts-te}$

(%i89) tsGBM_numeric_values:[ts=-3.5*10^9*yr,m(ts)=1,te=0,m(te)=50*10^6];

(%o89) $[ts=-3.5\;10^9\,yr, \mathrm{m}(ts)=1, te=0, \mathrm{m}(te)=50000000]$

(%i90) B_of_tsGBM,tsGBM_numeric_values;

(%o90) $B=\frac{2.8571428571428571\;10^{-10}\,\log(50000000)}{yr}$

(%i91) B_in_yr:ev(%,numer);

(%o91) $B=\frac{5.0650095895406915\;10^{-9}}{yr}$

(%i92) yr_vs_sec:yr=365.25*24*60*60*sec;

(%o92) $yr=3.15576\;10^7\,sec$

(%i93) B_in_sec:B=rhs(B_in_yr),yr_vs_sec;

(%o93) $B=\frac{1.6050046865226414\ 10^{-16}}{sec}$

GBM case.

(%i94) GBM_case:[m(p)=A*%e^(B*p),sL=sqrt(2*B)];

(%o94) $[\mathrm{m}(p)=A\ \%e^{p\ B}, sL=\sqrt{2}\ \sqrt{B}]$

(%i95) final_GBM_sigma_and_mu_of_p:final_sigma_and_mu_of_m_of_p,GBM_case;

(%o95) $[\sigma=\frac{1}{\sqrt{2}\ \sqrt{\pi}\ A}, \mu=\frac{1}{4\ \pi\ A^2}-p\ B]$

This completes the study of the Peak-Locus Theorem.

ENTROPY of the Running b-lognormal

Shannon ENTROPY of the Running b-Lognormal in bits.

(%i96) def_H:H=(log(sqrt(2*%pi)*sigma)+mu+1/2)/log(2);

(%o96) $H=\frac{\log(\sqrt{2}\ \sqrt{\pi}\ \sigma)+\mu+\frac{1}{2}}{\log(2)}$

(%i97) towards_def_H_of_m:def_H,final_sigma_and_mu_of_m_of_p;

(%o97) $H=\frac{\log\left(\frac{\%e^{\frac{p\ sL^2}{2}}}{\mathrm{m}(p)}\right)+\frac{\%e^{p\ sL^2}}{4\ \pi\ \mathrm{m}(p)^2}-\frac{p\ sL^2}{2}+\frac{1}{2}}{\log(2)}$

(%i98) H_of_m:distrib(radcan(towards_def_H_of_m));

(%o98) $H=\frac{\%e^{p\ sL^2}}{4\ \pi\ \log(2)\ \mathrm{m}(p)^2}-\frac{\log(\mathrm{m}(p))}{\log(2)}+\frac{1}{2\ \log(2)}$

(%i99) def_H_of_ts:H_of_ts=subst(ts,p,rhs(H_of_m));

(%o99) $H\_of\_ts=\frac{\%e^{ts\ sL^2}}{4\ \pi\ \log(2)\ \mathrm{m}(ts)^2}-\frac{\log(\mathrm{m}(ts))}{\log(2)}+\frac{1}{2\ \log(2)}$

NON-LINEAR Evo-ENTROPY (in bits) of the Running b-Lognormal

(%i100) def_NonLinearEvoEntropy_of_p:NonLinearEvoEntropy_of_p=(-rhs(H_of_m)+rhs(def_H_of_ts))

(%o100) $NonLinearEvoEntropy\_of\_p = \frac{\%e^{ts\ sL^2}}{4\ \pi\ \log(2)\ m(ts)^2} - \frac{\%e^{p\ sL^2}}{4\ \pi\ \log(2)\ m(p)^2} - \frac{\log(m(ts))}{\log(2)} + \frac{\log(m(p))}{\log(2)}$

(%i101) NonLinearEvoEntropy(p):=(-H(p)+H(ts));

(%o101) $NonLinearEvoEntropy(p) := -H(p) + H(ts)$

(%i102) 'NonLinearEvoEntropy(ts)=NonLinearEvoEntropy(ts);

(%o102) $NonLinearEvoEntropy(ts) = 0$

EXACTLY LINEAR EvoEntropy for Geometric Brownian Motion starting at ts.

This is the EXACTLY LINEAR MOLECULAR CLOCK.

LINEAR EvoEntropy for the tsGBM case.

(%i103) def_linearEvoEntropy_of_p_for_tsGBM:def_NonLinearEvoEntropy_of_p,tsGBM_case,def_Ns_v

(%o103) $NonLinearEvoEntropy\_of\_p = \frac{\log(Ns\ \%e^{(p-ts)\ B})}{\log(2)} - \frac{\%e^{2\ p\ B - 2\ (p-ts)\ B}}{4\ \pi\ \log(2)\ Ns^2} + \frac{\%e^{2\ ts\ B}}{4\ \pi\ \log(2)\ Ns^2} - \frac{\log(Ns)}{\log(2)}$

(%i104) EvoEntropy_of_p_for_tsGBM:linearEvoEntropy_of_p_for_tsGBM=factor(radcan(rhs(def_linearE

(%o104) $linearEvoEntropy\_of\_p\_for\_tsGBM = -\frac{(ts-p)\ B}{\log(2)}$
